# Iron Deficiency in Pulmonary Hypertension—Prevalence, Impact on Prognosis and Disease Burden in Pulmonary Arterial Hypertension and Pulmonary Hypertension Related to Hypoxia: A Review

**DOI:** 10.3390/ijms27052333

**Published:** 2026-03-02

**Authors:** Agata Krystyna Ołdakowska, Karol Adam Kamiński, Katarzyna Ptaszyńska

**Affiliations:** 1 Department of Cardiology and Internal Medicine, University Hospital of Bialystok, 15-276 Bialystok, Poland; agata.oldakowska@sd.umb.edu.pl (A.K.O.); karol.kaminski@umb.edu.pl (K.A.K.); 2 Department of Population Medicine and Lifestyle Diseases Prevention, Medical University of Bialystok, 15-276 Bialystok, Poland

**Keywords:** pulmonary arterial hypertension, right ventricular failure, hypoxia, iron deficiency

## Abstract

Pulmonary hypertension (PH) is recognized for being a severe, chronic phenomenon that necessitates a careful multidisciplinary approach. Its frequent coexistence with multiple comorbidities highlights the need for tailored decision-making concerning treatment towards not only certain PH subtypes but also towards each individual patient as well. Pulmonary arterial hypertension (PAH) management has undergone extensive development, which enabled patients’ life expectancy to be prolonged. The targeted treatment made a significant contribution to the improvement of the patients’ quality of life, thereby reducing the illness burden. However, apart from the administration of drugs in the course of PAH, there is also the field for determining and addressing modifiable factors, which may influence everyday life and the final outcome of these individuals. Taking into consideration the fact that iron deficiency (ID) is the most prevalent nutritional deficit worldwide and that there exists a well-established, scientifically supported correlation between ID and the outcome and prognosis of left heart failure patients, multiple studies were conducted in order to verify a possible connection between ID and right heart failure as well. Indeed, the crossroads of iron and PAH, PH related to hypoxia, and pathophysiological mechanisms linking pulmonary vasculature and ID have been eagerly investigated over recent years. Therefore, research provided a considerable amount of data in this area, emphasizing the potential usefulness of iron homeostasis to serve as a prognostic factor. Nevertheless, due to extensive exploration of this matter, several issues have arisen that demand further study and clarification, with the use of a proper ID definition being one of the most crucial. Herein, we present a concise review of the most up-to-date literature regarding iron’s homeostasis and pulmonary vascular bed through the prism of PAH and PH related to hypoxia.

## 1. Introduction

### 1.1. Pulmonary Hypertension

Pulmonary hypertension (PH) is a relatively rare (the overall incidence in the general population is estimated as 1%), progressive, and lethal disease [1,2]. In accordance with the latest guidelines by the European Society of Cardiology (ESC), PH is diagnosed when an increase in the mean pulmonary artery pressure (mPAP) over 20 mmHg at rest in direct measurement during right heart catheterization (RHC) is observed [3]. These criteria replace the former definition—over 25 mmHg, which was used in a significant part of previous studies concerning PH. Notably, echocardiographic measurements, in spite of not being suitable for making a definite diagnosis, are still serving as a valuable tool in screening for people who are at risk of developing PH due to the relevant accessibility and non-invasiveness of this method. Moreover, the values estimated echocardiographically correspond quite well with the ones obtained during the RHC [4,5]. Nonetheless, the latter remains a gold standard technique for diagnosing PH, as it enables a more precise and accurate measurement of pressures inside the heart and main vessels and allows us to diagnose which certain subtype of PH a patient suffers from [3,6].

**Figure 1:**
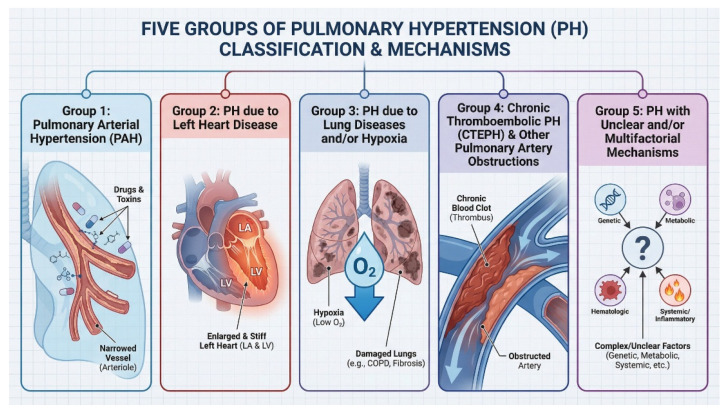
The presentation of five subtypes of PH. First PH subtype—PAH, represented by narrowed vessels (arterioles in the lungs)—second subtype complicating left heart disease, third subtype linked to lung diseases and hypoxia, fourth subtype—CTEPH linked to obstruction of arteries caused by blood clot formation, and fifth—multifactorial PH. The graphic was created with the use of AI [13].

There are two systems of PH’s classification, one of which comprises five different subtypes, involves the cause of the pressure increase in the pulmonary bed, and is more eagerly used in clinical practice (Figure 1) [3]. The first subtype, pulmonary arterial hypertension (PAH), comprises a specific group of patients that consists of the constellation of distinct morbidities that are linked due to the pathophysiological similarity. PAH covers, amongst others, congenital heart diseases (CHD), also complicated by Eisenmenger Syndrome (ES), idiopathic PAH (iPAH), hereditary PAH (hPAH), and PAH related to connective tissue disorders (especially systemic sclerosis). Additional criteria created to diagnose PAH and to enable distinguishing it from other PH types are the pulmonary vascular resistance (PVR), when greater than two Wood Units (WU), and pulmonary artery wedge pressure (PAWP) 15 mmHg or lower [7]. PAH is often in the scope of interests of many researchers, probably at least partially owing to its extremely unfavorable prognosis, i.e., before the targeted treatment was implemented, median survival had rarely exceeded three years, as in many cancers at an advanced stage [8]. The PAH-specific treatment encompasses, amongst others, the following: endothelin-1 (ET-1) receptor antagonist, phosphodiesterase 5 inhibitors, exogenous nitric oxide (NO), soluble guanylate cyclase stimulator, and prostacyclin (PGI_2_) receptor agonists [3].

The second subtype of PH is the one that complicates the diseases of the left heart. This is the most common type of PH worldwide, and iron supplementation’s role in the field of left heart failure (LHF) is well established [9,10,11]. On account of the extensiveness of this matter, discussing it exceeds the range of this review. Albeit, it is partially by dint of the research conducted on the LHF and ID and the promising results of iron restoration in this area, which encouraged investigators to perform similar studies on the influence of ID and iron supplementation on the right heart failure (RHF) as well.

Iron and its deficiency are claimed to play a pivotal role in the third type of PH—the one resulting from hypoxia, hypoventilation, and lung diseases, of which theme we willingly encompass in the pages of this article. There is a common point of vital importance—hypoxia inducible factor (HIF), which provides a strong linkage between these conditions [12].

The fourth type of PH—chronic thromboembolic pulmonary hypertension (CTEPH)—and the fifth subtype of PH lie beyond the scope of this review.

The other system of PH classification is based on the hemodynamics (Table 1): precapillary, postcapillary, and combined pre- and postcapillary PH. Precapillary PH is usually represented by the first, third, and fourth subtypes, and isolated postcapillary PH often coexists with LHF, whereas mixed pre- and postcapillary PH is represented mainly by PH complicating systemic disorders and instances of co-occurrence of chronic obstructive pulmonary disease and left heart disease [14].

PH is a challenging illness since it is troublesome to diagnose, partly because of its rarity and also to some extent due to its nondistinctive symptoms such as exercise-induced dyspnea, fatigue, palpitation, fainting, and weight gain caused by fluid retention—any of which may indicate many different diseases [15,16]. Moreover, despite the improvement in PH’s treatment over the past decades, it is still associated with remarkable deterioration in everyday functioning and poor prognosis, which in some cases leads to the necessity of lung- or heart–lung transplantation as a final treatment [17,18].

### 1.2. Iron and Its Deficiency

**Figure 2:**
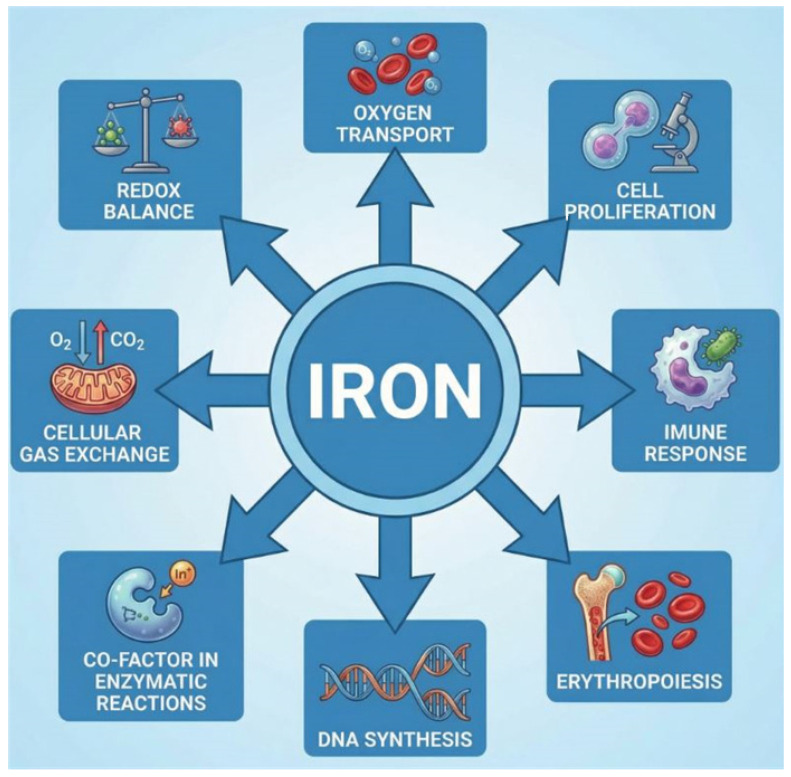
The presentation of the diverse roles of iron in the human body. The graphic was created with the use of AI [13].

Iron is an essential element of life and plays a multifaceted role in the human body (Figure 2) [19]. The depletion of iron stores is well-known to deteriorate patients’ condition in many diseases, such as chronic obstructive pulmonary disease (COPD) or acute and chronic heart failure (HF), amongst others [10,20,21]. Furthermore, ID is reported to increase the odds of cerebrovascular events and is an established prognostic factor in acute ischemic stroke [22,23]. On the other hand, iron is necessary for many pathogens’ growth, and it also takes part in the reactive oxygen species formation, and via these paths, it promotes inflammatory processes and oxidative stress [24].

In circulation, iron is bound to the transferrin, whose role is to protect cells and tissues from the toxic effect of unbound iron, and in this form, iron is transported to the target cells and endocytosed via TfR1 (transferrin receptor protein 1) on the cell surface. The transferrin saturated with iron (TSAT) at the level of 20–45% is considered to be physiological, while TSAT over 80% reflects severe iron overload, which is associated with the risk of tissue toxicity since iron occurs in the unbound state in large amounts as well [25].

ID may be absolute or functional. The main difference between these two is that the absolute ID occurs in the lack of stored iron secondary to inadequate dietary intake, malabsorption or chronic blood loss, whilst functional ID arises subsequently to the decreased concentration of the circulating iron in the presence of the adequate amount of iron in its magazines; for instance, in terms of persistent inflammation, parasitic infection, and cancers [26]. Moreover, there are three phases of ID. Anemia related to ID is the last stage, characterized by a low hemoglobin concentration reflecting depleted iron stores, low serum iron, and TSAT. The two anticipatory stages are the latent stage, associated with low ferritin, serum iron, and TSAT, and the pre-latent stage, characterized solely by the reduced iron stores [27]. ID may be symptomatic even in the absence of anemia; therefore, an active search for ID and treatment should be the standard of care [28]. The factor emphasizing the importance of functional ID in clinical management of patients is the fact that levels of zin-protoporphyrin are high in iPAH, and this particle is strongly correlated with disease severity. Zinc is a molecule competing with iron for the binding site. Therefore, when iron levels decrease, zinc takes its place in the active sites, as for instance in hemoglobin, and therefore high levels of zinc–protoporphyrin reflect the state of functional ID [29].

### 1.3. ID Definitions

The great variety of definitions and forms of ID in the studies concerning PH causes significant differences in the proportion of patients who are defined as iron-deficient. Furthermore, distinguishing between anemia, which is caused by ID, and that deriving from chronic diseases is strenuous if relying just on standard iron homeostasis parameters, such as TSAT or ferritin, as both of them are affected by inflammation. Ferritin is an acute phase protein that is increased, while transferrin is a reverse acute phase protein that diminishes in terms of the inflammatory process, which in turn generates falsely elevated TSAT. Therefore, there is a need for applying more specific biomarkers, which are less prone to be influenced by confounding factors.

The research by Sonnweber et al. concentrated on the issue of using the proper ID diagnostic criteria in PH, as depending on the ID criteria applied, the percentage of PH ID patients might vary from 11% to 75% [30]. Consequently, the definitions, which include both true and functional ID, carry the risk of potential overtreatment, which may cause hyperviscosity symptoms, a hypercoagulable state and other complications resulting from iron overload and its toxicity on tissues, while strict definitions based on TSAT < 16% and ferritin levels < 30 μg/L depict only patients who are profoundly iron deficient, and the benefits of iron replacement in these individuals are undeniable (Table 2). Therefore, definitions which are based on the soluble transferrin receptor (sTfR) are considered to be the most sensitive and specific determinants of ID even in the presence of interrupting points. Particularly, the sTfR/log ferritin (sTfR-F index) is an appreciated parameter of the iron homeostasis, as it reflects the tissue demand for iron as well. Furthermore, it was claimed to be more precise in presenting a linkage between ID and clinical outcome in comparison with previously described ID criteria [30]. Regardless of the definition used, it is clear that ID is a prevalent comorbidity in PH, and it may have a significant impact on the disease course. Albeit, it is the lack of standardized guidelines that makes the attempt to compare the impact of ID or its substitution on different patient populations arduous.

Despite the fact that ID was shown to be a negative prognostic factor, there are also studies that do not reveal such a correlation. For instance, in a study by Xanthouli et al., low ferritin and low serum iron correlate neither with time to clinical worsening nor survival, albeit these factors are still eagerly used in clinical practice to assess iron homeostasis and to decide whether to start iron supplementation or not [31]. Of note, iron is an extremely fluctuant particle, and its serum concentration cannot serve as a reliable determinant as it is dependent from the time of day and food intake and could vary as much as 30% during the day [32].

In the pages of this review article, the results of research concerning ID and PH are gathered. We mention ID as a comorbidity in LHF, concisely presenting known pathomechanistic linkages between ID and the pulmonary vascular bed in PAH and hypoxia-related PH. What is more, there are also collected observational studies, which provide information about the prevalence and potential usefulness of iron homeostasis-related parameters to serve as prognostic factors and results of experimental studies in ID and PH in rodent models, as well as trials regarding iron supplementation in different PAH subtypes.

## 2. ID and LHF

ID in LHF, especially in acute HF, is a more common phenomenon than in the case of PAH. ID is shown to be associated with worse outcomes independently of anemia [33]. A few investigations concerning ID revealed that the depletion of iron stores is related to impaired quality of life, which is caused by fatigue and lower exercise capacity. Additionally, ID is accompanied by worse clinical outcomes, i.e., more frequent hospitalizations and shortened life expectancy [34,35,36].

At the cellular level, ID affects myocardium directly, causing the dysfunction of mitochondria and restrained ATP production, which afterwards leads to oxidative stress and the impairment in left ventricular function [37,38,39]. Moreover, iron supplementation, but not the correction of anemia itself, exerts a positive influence on HF. Consistent with this, there are trials that validate that iron restoration not only causes improvement in the field of exercise tolerance and quality of life but also reduces hospitalizations related to HF [40,41,42,43,44,45,46] (Table 3).

## 3. A Short Insight into the Molecular and Mechanistic Links Between PAH and Iron Homeostasis

PAH is a thoroughly explored illness in the field of coexisting ID. There are a few debated pathways of how PAH and ID may interplay. Pathophysiology of PAH comprises the remodeling that takes place in the pulmonary vascular bed, inflammatory activation, and imbalances in vasoactive mediators [47].

**Figure 3:**
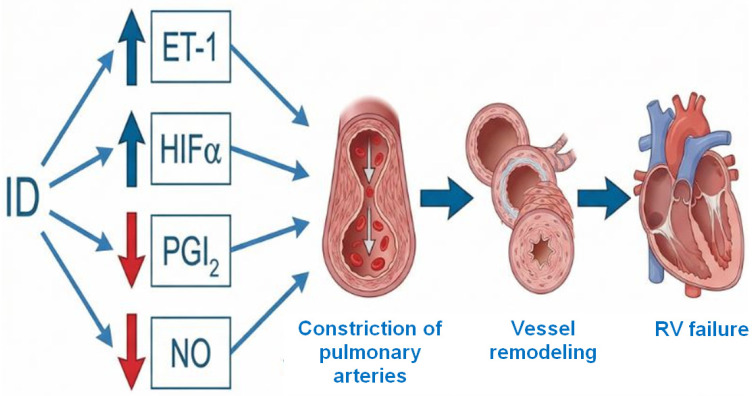
The impact that ID exerts on the pulmonary vascular bed via vasoactive molecules. ET-1—endothelin-1, HIFα—hypoxia inducible factor alpha, NO—nitric oxide, PGI_2_—prostacyclin, RV—right ventricle. The graphic was created with the use of AI [13].

The dysregulation in vasoactive molecule homeostasis, i.e., chronic impairment of potent vessel relaxants production, nitric oxide (NO) and prostacyclin (PGI_2_), together with prolonged overexpression of angiospastic endothelin-1 (ET-1), is postulated to enhance the endothelium dysfunction with subsequent imbalance in pulmonary arteries’ tone and thereby to initiate the vicious circle of phenomena occurring in PAH [48]. Notably, all these particles are associated with iron homeostasis (Figure 3) [49,50,51].

ID promotes changes in vascular tension and pulmonary vessel remodeling, which involve the proliferation of not only pulmonary artery smooth muscle cells but also pulmonary artery endothelial cells, fibroblasts, myofibroblasts, and pericytes. Indeed, the arteries’ smooth muscle layer lying underneath the damaged endothelium is more prone to further injury and the influence of the proliferative cytokines, which results in the hypertrophy of intima and media in small pulmonary arteries, with subsequent fibrosis and formation of plexiform vascular lesions—key features of PAH [52]. Additionally, in PAH, a persistent low-grade inflammation is observed, which might be associated with the elevation of serum interleukin-6 (IL-6) and a subsequent increase in hepcidin levels [53].

**Figure 4:**
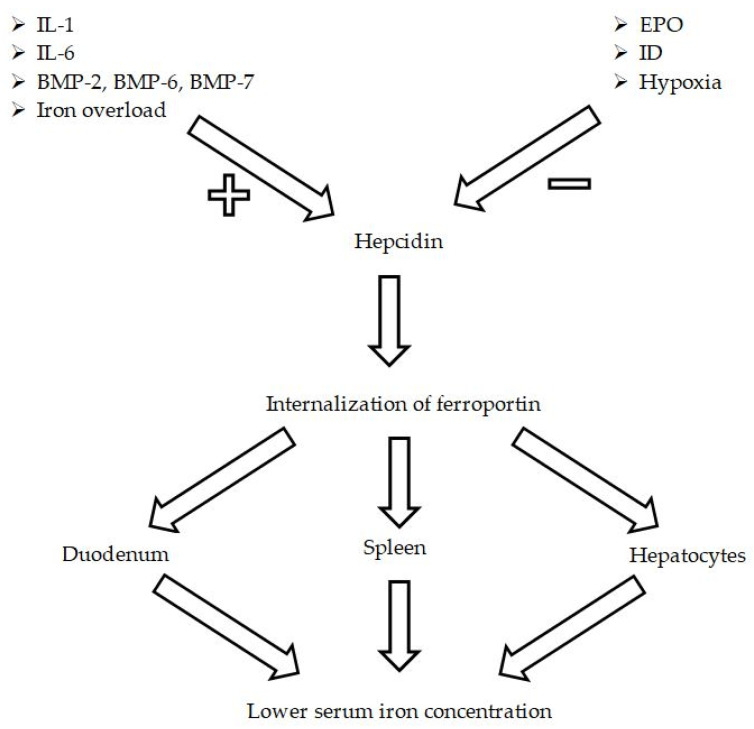
Hepcidin-ferroportin axis. The presentation of factors influencing hepcidin expression. Hepcidin causes the internalization of ferroportin, which normally enables iron release to the circulation.

Hepcidin—a small acute phase protein produced by the liver—prevents iron absorption in the duodenum by binding to the ferroportin, the only known receptor for iron, which enables its release to the bloodstream from enterocytes and from iron storage cells in the liver, the spleen, and bone marrow [54]. This mechanism emerged so as to enable mammals to fight against infections, as pathogenic microorganisms need iron to survive. Nonetheless, other possible causes of inflammatory activation encompass chronic diseases, such as for instance, LHF or PAH [55]. Hepcidin itself is upregulated by many factors, including interleukin 1 (IL-1), IL-6, bone morphogenetic protein 6 (BMP-6), and iron overload, while it is downregulated by erythropoietin (EPO), ID, and hypoxia (Figure 4) [56]. Taking into consideration the bone morphogenetic protein pathway, BMP-6 binds to its receptor, which further autophosphorylates and causes hepcidin coding gene transcription [57]. Therefore, bone morphogenetic protein receptor 2 (BMPR2) mutation seems to be an interesting issue in the aspect of hepcidin regulation, iron homeostasis, and PAH, as more than 70% of patients with hPAH were found to be carriers of this mutation, as well as 20% of iPAH individuals [58]. Furthermore, hPAH patients with this mutation were younger at the time of diagnosis, presented with worse hemodynamics, a higher rate of lung transplantations, and mortality of all causes [59]. Contrarily, despite the fact that IL-6 is claimed to be one of the factors upregulating the production of hepcidin, their serum concentrations were also reported by some investigators not to correlate, and others conclude that hepcidin might be upregulated by ID to a greater extent than by the inflammatory process and IL-6. Nevertheless, the measurement of IL-6 still might be advised as its higher concentration is related to mortality in PH [60].

Additionally, in terms of ID, hepcidin levels are usually low, and consequently, this phenomenon should be observed in iron-deficient PAH patients. Therefore, there actually is a subgroup of patients with very low hepcidin reflecting deep ID—these individuals may potentially benefit from oral treatment. Contrarily, another subgroup of patients presents with abnormally high hepcidin, which might further potentiate ID by inhibiting iron absorption [55,61].

Finally, PH is indispensably linked with the impairment of the right ventricular (RV) function, as the latter is a key in the matter of adaptation to the increased afterload, and its deterioration serves as a prognostic factor in PH. ID may impair RV function by disturbing its contractile abilities and reducing vulnerability to changes in PVR. ID, which occurs inside RV cells, may contribute to the ischemia and further potentiate RV dysfunction, leading to adverse outcomes in PH [30,62,63].

As presented above, there are several common points of iron and cardiovascular homeostasis, including PH; therefore, iron and its deficiency in this disease’s course need further research to comprehensively investigate this issue.

## 4. ID in Animal Models with PH

Incontrovertibly, the most inconsistent data arose from experimental models of PH. Lakhal-Littleton et al. conducted a study on mice, which intelligibly revealed that ID inside pulmonary arterial smooth muscles alone was able to cause PAH due to the increased expression of ET-1. Furthermore, the administration of iron and an ET-1 antagonist prevented the increase in pulmonary pressures [51]. Additionally, Cotroneo et al., who investigated rats that were receiving an iron-deficient diet for four weeks, provided results enabling them to conclude that this diet resulted in ID, which in turn contributed to profound pulmonary vascular remodeling and a subsequent increase in pulmonary pressures. However, even more importantly, these changes were reversible by dint of iron supplementation [64].

On the contrary, Naito et al. investigated mice that were exposed to four-week-lasting hypoxia and randomized them into two groups—receiving either an iron-restricted or a normal diet. In these mouse models, the reduction in available iron had a moderate protective role against pulmonary remodeling [65]. Similarly, in another study by these authors, rats received monocrotaline so as to induce PH, and an iron-restricted diet diminished the pulmonary vascular remodeling and RV hypertrophy [66]. Wong et al. revealed that the infusion of deferoxamine, an iron chelator, attenuated the pulmonary vascular remodeling and pulmonary hypertension induced by chronic hypoxia [67].

These data, although diverse, suggest iron’s entanglement in processes occurring in pulmonary vessels. Nevertheless, ongoing research on the matter should be encouraged so as to clarify the ambiguities.

## 5. ID and Outcome in Patients with PAH of Different Origins

The previous research in PAH provide consistent outcomes: regardless of the subtype of PAH, ID is reported to correlate with the presence of the more exaggerated disease symptoms, worse hemodynamics (defined as higher mPAP, mean right atrial pressure, lower cardiac index), decreased 6-min walk distance (6MWD), worse functional capacity measured during cardiopulmonary exercise test (CPET) and higher NT-proBNP concentration. Furthermore, ID coexists with a reduced quality of life, a more severe NYHA/WHO functional class (FC), and even increased mortality [68,69,70]. Despite the fact that ID is claimed to show a predictive value for mortality or disease exacerbation, some investigations showed ID is neither an independent nor significant predictor of survival in PH, while it is associated with worse exercise capacity [71].

On the contrary note, there is a Jackson Heart Study in which investigators desired to determine whether there is an association between ID, defined as low ferritin concentration, and PH in the general population. Consequently, they did not report such a correlation, although it may emanate from the fact that pressures were estimated echocardiographically, with pulmonary artery systolic pressure (PASP) over 40 mmHg being the determinant of PH’s diagnosis, and additionally ID was diagnosed relying on ferritin values only, which excludes individuals with functional ID [72]. The part of the PH population with functional ID is not less important and should not be overlooked, as even the functional ID is postulated to be recognized and treated as a mere condition by some researchers, as these patients might benefit from iron supplementation [28]. Moreover, some researchers suggest that an adequately long period of time might be necessary to allow ID to cause the increase in the pulmonary pressures, or contrarily, pathophysiologic mechanisms activated in the pulmonary vascular bed may also need time to cause ID.

### 5.1. ID in Scleroderma

The majority of data is consistent that ID is a prevalent comorbidity in PAH, although connective tissue disease–PAH individuals are probably the most prone to develop ID due to the more prominent impairment of iron absorption in the intestine secondary to the connective tissue dysfunction [73]. In the scleroderma-related PAH, Ruiter et al. studied the prevalence of ID in the course of this illness, which turned out to be three times higher in scleroderma complicated by PAH than in scleroderma itself, which emphasizes the connection between ID and pulmonary vasculature. ID contributes to the impaired exercise capacity (defined as 6MWD and CPET) and compromised survival in both ID-suffering groups, while lower 6MWD correlated with higher sTfR values [74].

### 5.2. ID in CHD

Another subgroup of patients, which requires particularly insightful observation, is the one comprising individuals suffering from CHD-related PAH with inclusion of patients with Eisenmenger Syndrome (ES), whose blood oxygenation is permanently decreased because of the mixing of oxygenated and deoxygenated blood due to a right-to-left shunt, resulting from abnormalities such as atrial, ventricular or atrioventricular septal disease to name a few, which in turn enhances the erythropoietin production [75,76]. Nevertheless, erythropoiesis cannot be efficient in an iron-deficient environment. In such a case, red blood cells are more prone to degradation and lysis, and their aggregation ability is decreased, which perpetuates the vicious circle of ID [77]. Furthermore, for the same hematocrit, individuals with enough iron stores present with bigger red blood cells that containing more hemoglobin compared to ID patients [78]. In this subset of patients, iron plays a significant role. Research on ES patients by De Bruaene et al. supports the thesis that ID, amongst others, is a factor contributing to shorter event-free survival and higher mortality of all causes [79]. Notably, ES patients usually present with additional risk factors, which may enhance the chances of developing ID, such as the use of oral anticoagulation and phlebotomies due to hyperviscosity symptoms. Therefore, patients who receive anticoagulation and phlebotomies should be rigorously monitored in terms of ID [80].

Of note, CHD-PAH patients are usually younger than those representing other PH subgroups, which emphasizes the fact that more prosaic issues, such as menstruation, may be an important factor that contributes to ID as well, which should not be overlooked, as they are potentially straightforward to prevent or cure. The frequency of ID in PAH menstruating women exceeded the frequency of ID in PAH postmenopausal women and PAH men, and what is more, PAH was related to a higher proportion of iron-deficient individuals than CTEPH when comparing whole cohorts, as well as with the consideration of each singular subgroup [81].

### 5.3. Novel Biomarkers of ID

Apart from the parameters that are in a quite obvious way related to iron homeostasis, i.e., serum iron concentration, serum ferritin, transferrin, TSAT, and sTfR, there are several less-known markers that reflect iron status indirectly and may serve as novel biomarkers of ID. Some of them have the potential to outweigh conventional measurements in predicting outcome and survival.

Hypochromic red cells (HRC), presenting over 2% of all erythrocytes, in a study by Xanthouli et al. is a factor related to the shorter time to clinical worsening, worse survival at the time of diagnosis and it was the only variable that maintained significance after a year of follow-up. Notably, only 64% of patients presenting with HRC > 2% were also iron deficient as determined with the use of ferritin. Another issue is that HRC is the early marker of anemia, and additionally, it reflects iron bioavailability in the previous 120 days [28]. Therefore, it may serve as an index of response to the treatment, which broadens its possible usefulness in PAH [31]. Moreover, it was reported to be an independent predictor of mortality in systemic sclerosis patients who were screened for PH [82].

Another factor potentially related to the disease severity might be the red cell distribution width, which, like the percentage of red cells that are hypochromic, is a marker of subclinical ID. It was reported to be associated with higher mPAP and shorter 6MWD, independently, and the correlation with mortality was stronger than in the case of other well-established markers, such as, for instance, NT-proBNP [83,84].

Interestingly, the ratio of immature to total reticulocytes in peripheral blood assessed during cytometry is thought to serve as a sensitive marker of erythropoiesis, and it turned out to be more efficient than TSAT or sTfR in terms of correlating with PAH hemodynamic severity [85].

All these data together suggest that there is a potential to find novel, complementary markers of iron homeostasis which might be useful in predicting clinical outcomes and survival in PAH.

## 6. Iron Restoration in ID in PAH Patients

### 6.1. Challenges of Certain Routes of Iron Administration

**Figure 5:**
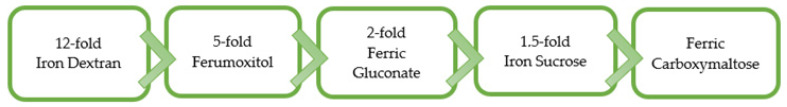
The figure demonstrates the risk of anaphylaxis of intravenous iron solutions when compared to FCM.

Considering research that focused on iron’s restoration in ID PAH patients, they depicted that oral as well as intravenous iron supplementation was safe in these cohorts. Despite the fact that oral iron supplementation carries a reduced risk of anaphylaxis when compared to the parenteral route, to the best of our knowledge, no such detrimental effect of intravenous iron in PAH was reported. What is more, the odds of developing an anaphylactic reaction vary with the use of a certain iron solution; iron dextran is claimed to be associated with the highest risk, while the following were related to decreasing the possibility of developing anaphylaxis: ferumoxytol, ferric gluconate, and iron sucrose. Ferric carboxymaltose is considered to be the safest parenteral solution according to Dave et al. (Figure 5) [86].

Despite the fact that oral iron is related to a lower anaphylaxis risk, it presents other detrimental effects: gastrointestinal side effects, the possibility of impaired absorption, and decreased efficacy in comparison with the intravenous route of restoration (Table 4). On the other hand, oral supplementation is cost-effective, and the effects are more promising when oral iron is administered less frequently; for instance, every other day instead of three times daily [87]. All in all, the intravenous route of iron’s administration serves as the more efficient way of iron restoration and to improve the patient’s clinical condition and outcome. Albeit, it is worth considering that these data constitute a strong basis for further investigation on whether oral iron supplementation may serve as the first-line therapy in mild-to-moderate ID or as a maintenance therapy after intravenous infusion, as recurrence of ID was reported to be frequent in PAH patients [88,89].

### 6.2. Results of Iron Restoration in PAH Patients

Oral iron supplementation is claimed to be less effective as it may not be able to adequately replenish iron magazines—even up to about 50% of patients might not respond to the treatment, as it was reported in the study by Ruiter et al. [70]. Contrarily, another two studies on oral iron supplementation in PH provide data that not only did iron status improved, but so did 6MWD. What is more, one of these studies showed significantly lower NT-proBNP levels after iron restoration and a decrease in RV diameters [90,91]. Moreover, the WHO functional class (FC) improved in some individuals, while its worsening was not observed. Each from abovementioned studies conducted follow-up visits after 12–16 weeks, hence a long-term study would also be appreciated so as to assess the possible fluctuations in the levels of iron [70,90,91].

In terms of intravenous iron restoration, all studies are consistent that it is an efficient approach in re-establishing iron homeostasis, and iron infusion is devoid of severe adverse effects. Albeit, the majority of studies are neither placebo-controlled nor randomized and are preponderantly open-label, comprising small patient cohorts (Table 5). Actually, there is only one randomized placebo-controlled trial, which failed to depict any other improvement in patients’ condition (in fields of exercise capacity, cardiopulmonary hemodynamics, quality of life), apart from iron storage, which was indeed replenished. However, the vast majority of patients presented with II WHO FC, and it should not be unmentioned [92]. Moreover, in the study by Ruiter et al., 6MWD remained unchanged; however, the exercise time in CPET was longer, and aerobic capacity improved as well. These changes were postulated to stem from better oxygen binding capacity at the level of skeletal muscle since cardiac function was unchanged [93]. Similarly, Biener et al. report no significant changes in 6MWD, WHO FC, NT-proBNP, and quality of life, with fatigue being the only variable to improve and to reach statistical significance [89]. Contrarily, Viethen et al. depict that 6MWD as well as quality of life were significantly better after iron infusion in iron-deficient PAH patients, while emphasizing treatment’s safety [73].

The study by Kramer et al. was a long-term trial with the inclusion of iron-deficient individuals as well as a control group [88]. This research, apart from showing that after iron supplementation, exercise capacity (6MWD) and the WHO FC improved, ESC risk and hospitalization rate decreased, also points out that even after intravenous iron infusion, patients need watchful screening for ID as this condition tends to recur. Additionally, according to Biener et al., patients who were not iron-deficient at baseline and served as a control group developed ID later on, which underlines the necessity of careful observation of iron status in all PAH patients [89].

Iron-deficient CHD patients with lower oxygen saturation are at risk of developing hyperviscosity symptoms due to enhanced yet insufficient erythropoiesis and therefore need caution during iron restoration [94]. However, in the subset of patients with CHD-PH and/or Eisenmenger Syndrome, iron restoration was related to improvement in the quality of life, 6MWD, and furthermore, iron infusion due to cautious administration was not reported to cause any hyperviscosity syndrome, which is extremely relevant in this cohort of PAH patients [53]. Taking into consideration the foregoing research, it might be currently advised to restore iron with low doses with careful supervision, considering ID as a factor impairing oxygen carrying capacity of these individuals as well.

## 7. ID in the Third Subtype of PH

**Figure 6:**
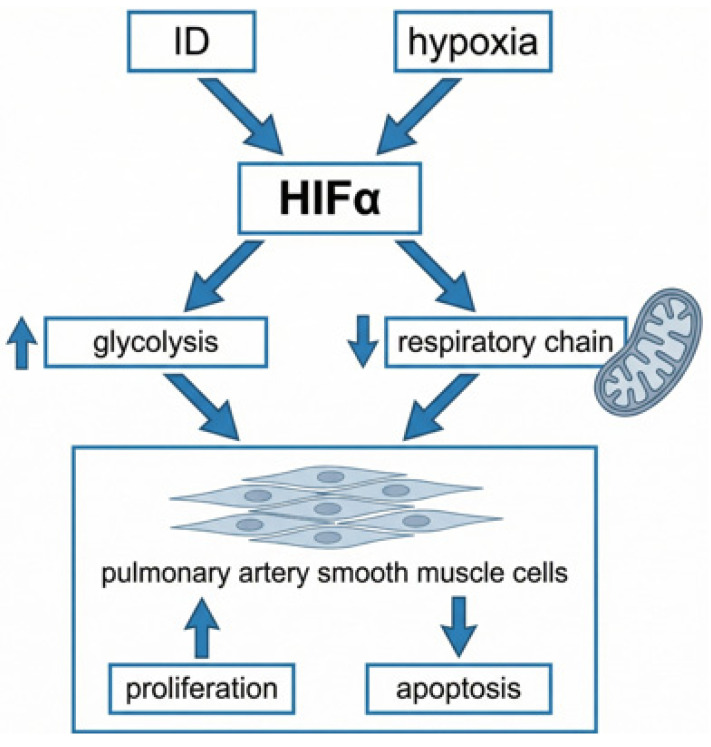
The augmentative effect of ID and hypoxia on pulmonary artery smooth muscle cells. The graphic was created with the use of AI [13].

PH related to hypoxia and lung diseases is the second most common subtype of PH. The main cause of pulmonary vasoconstriction and remodeling in this condition is hypoxia. Notably, in pulmonary vasculature, hypoxia causes vessels’ constriction—the exact opposite effect than the one observed in the systemic vascular bed, where it acts as a vasodilator [95]. ID is known to mimic hypoxia and to worsen its detrimental effects, while iron supplementation reduces pulmonary vascular constriction and alleviates PH [96]. The impact of hypoxia on the pulmonary vascular bed is mediated via HIF pathways. In murine models, hypoxic stabilization of HIF-alpha causes proliferation, migration, and hypertrophy of pulmonary artery smooth muscle cells, leading to PH. The HIF-α particles are strongly correlated to iron, i.e., the depletion of iron regulatory protein-2 was found to induce the expression of Hif1α as well as Hif2α, thereby intensifying glycolysis and diminishing the expression of genes related to iron-sulfur cluster and electron transport chain, which altogether contribute to debilitating the process of mitochondrial respiration (Figure 6) [97].

**Figure 7:**
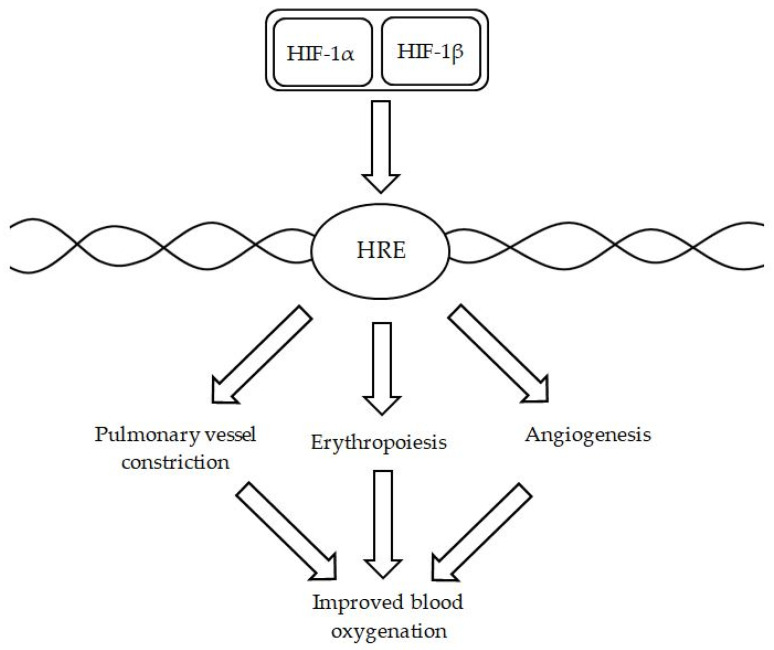
A simplified presentation of the influence of heterodimeric HIF particle on hypoxia-responsive elements (HRE) and further phenomena leading to the improved oxygenation of blood.

Furthermore, biochemically, HIF inactivation takes place in the presence of oxygen, and iron is necessary as an obligate cofactor. In a physiological, adaptive scenario, during hypoxia, HIF is a direct as well as indirect transcriptional factor for hundreds of genes, and as a heterodimeric particle made up of HIF-alpha and HIF-beta, it binds to hypoxia-responsive elements and affects transcription of genes, which are responsible for erythropoiesis and angiogenesis, enabling better oxygenation (Figure 7) [98]. Nevertheless, PH is not a physiological condition, so these actions, instead of being beneficial, often contribute to pathologic responses to hypoxia and disease progression—sustained excessive vasoconstriction and remodeling of small pulmonary arteries. Furthermore, HIFα and ET-1 appear to be factors involved strongly in PH related to chronic obstructive pulmonary disease (COPD), as Zhang et al. investigated these individuals and concluded that these particles presented a positive correlation with pulmonary pressures, whereas NO correlated inversely [99]. Therefore, upregulation of HIF secondary to ID could augment hypoxic pulmonary vasoconstriction. These mechanisms show beyond doubt that patients suffering from the third type of PH are uniquely vulnerable to the detrimental effects of ID.

Highlighting the importance of iron in the pulmonary bed, intravenous infusion of deferoxamine, a known iron chelator, is reported to cause an increase in PASP, and there was a correlation between individual response to hypoxia and iron chelation [100]. Similar findings were presented in the research by Smith et al., in which patients were divided into two subgroups receiving either intravenous iron or deferoxamine before being exposed to eight-hour-long hypoxia. The infusion of iron was found to have a protective effect and alleviated the increase in PASP. Additionally, authors drew the conclusion that after the rapid rise in pulmonary pressures caused by the acute hypoxia, there follows the rise in a slower onset, which may result from the alteration in gene expression caused by HIF, and the latter process might be especially prone to the influence of iron [101]. Comparable outcomes provided another investigation in which the rise in PASP following exposure to hypoxia was 60% higher in iron-deficient patients than in iron-replete ones. Furthermore, intravenous iron administration attenuated the increase in PASP, which was more apparent in the iron-deficient group [96]. Smith et al. conducted another study, which provided evidence that iron replacement reverses the increase in PASP caused by hypoxia to a greater extent than the placebo. Moreover, intriguing was the fact that patients with chronic mountain sickness, characterized by polycythemia, hypoxemia, and PH, presented with permanently elevated baseline PASP and venesections, causing the decrease in available iron, which resulted in ID and a concomitant rise in PASP. Nonetheless, there was not observed any significant change in PASP after intravenous iron therapy which in turn allowed investigators to suspect that either the amount of iron removed was too large to be replenished by that certain amount of iron administered intravenously or iron supplementation in case of the chronic pulmonary remodeling does not reverse the influence of ID on PASP, at least in a fast manner [102]. It is noteworthy that in appropriate and inappropriate acclimatization to altitude, the levels of erythropoietin were high, while hepcidin was markedly elevated in individuals who showed poor adaptation and low in the case of better adaptation.

In summary, the abovementioned studies show beyond doubt that iron plays an important role in the third type of PH. Nevertheless, most of these studies present with one issue in common, which might be perceived as a drawback, namely, the data concerning the hemodynamics of the right ventricle and pulmonary arteries were obtained mainly via echocardiography, not RHC, while the latter is a gold standard in assessing pressures inside the heart and main vessels.

## 8. Ambiguities, Gaps in Evidence, and Future Directions for Following Investigations

The drawback of studies concerning PH and ID is the lack of use of standardized ID definitions. This fact precludes straightforward reasoning and hinders the actualization of guidelines—the possibilities to create a meta-analysis or even systemic review are therefore limited. However, the abovementioned studies have been accumulating steadily over the recent decades, and a couple of years ago, definitions based on sTfR were not recognizable enough [103]. Furthermore, the definition of PH used in a great number of PH investigations is the old one, when it was diagnosed relying on mPAP as much as or over 25 mmHg—this situation brings a risk that many patients who now would be considered as PH-suffering were not identified, and subsequently, the iron-deficient cohort could potentially be underestimated. Additionally, many studies on PAH were conducted on small groups of patients, albeit, it results from the rarity of the disease. What is more, small sample sizes in the studies regarding PAH patients impede attempts to outline potential differences in prevalence and actual prognostic value that ID has on different subsets of PAH, respectively. Further, the dissimilarities between responses to iron restoration in these smaller subsets of patients are also hard to determine.

Moreover, the variability of outcomes cannot be unmentioned—some studies show outstanding improvement in numerous aspects of life of ID PH patients after iron supplementation, while others are more skeptical. Additionally, some researchers claim ID is an independent and strong predictor of clinical outcome or survival, while others are not able to demonstrate these effects [103].

Herein, we presented a few drawbacks of the cited research, yet the investigators still presented valuable and interesting results and conclusions. There is a need for further studies due to the lack of randomization of the overwhelming majority of the former studies, and also so as to elucidate the issues that remain unclear, i.e., the use of a standardized ID definition that would later enable proper setting of patients selection criteria in case of iron restoration. Moreover, the adequate dosing and route of administration for each PH cohort needs further investigation, including in terms of long-term outcomes. All the mentioned concerns need to be evaluated in order to optimize treatment and management strategies and to create updated guidelines.

## 9. Materials and Methods

During the preparation of this manuscript, the authors used Artlist.io AI Image Generator Nano Banana 2 for the purposes of generating Figure 1, Figure 2 and Figure 3 and Figure 6. The authors have reviewed and edited the output and take full responsibility for the content of this publication.

## 10. Conclusions

PH is a severe condition characterized by unfavorable outcomes of plentiful individuals, and for that reason, there were numerous attempts to find factors that might improve patients’ quality of life and life expectancy. Researchers, encouraged by the promising results from LHF and ID, investigated the issue of ID in PH as well. It was revealed that ID is a prevalent, modifiable, prognostic factor in disorders concerning the pulmonary bed and maintaining homeostasis of this microelement is a relevant matter for improving survival, outcomes and patients’ quality of life.

However, due to the small sample size of available studies and their neither randomized nor placebo-controlled nature, there is a need for large multicenter placebo-controlled randomized trials. Currently, routine iron homeostasis assessment is advised, whereas iron restoration should be cautiously implemented and thoroughly monitored, especially in PAH related to CHD and in cyanotic patients in general. Moreover, it must not be overlooked that, in view of the fact that ID frequently coexists with PH, which actually consists of numerous conditions that vary in terms of pathophysiology, the approach should be tailored individually to a patient.

Hence, as it was presented above, undoubtedly there is a link between PH, pulmonary vasculature, and ID; however, whether ID contributes to some extent to the severity of PH or is merely a consequence of a poor general condition remains to be elucidated.

## 11. Key Points

- ID is a prevalent comorbidity in many diseases, including PH and LHF
- It is possible and frequently observed that ID affects patients’ outcome in cardiovascular disease independently of anemia, and in some studies, iron-related factors are reported to be an independent predictor of survival
- There is a need to use a standardized definition of ID. The sTfR-F index seems to be the pretender for this role
- Patients suffering from PH as well as PAH should be screened for ID
- Previous studies reveal that the intravenous route of administration is more efficient than oral in patients with HF, including those with PH
- On the basis of up-to-date research, iron restoration seems to be safe in PAH as well as PH patients, and there is evidence that it is beneficial in terms of functional capacity and quality of life
- The third type of PH is the one in which iron restoration may be the most beneficial
- There are a few widely available markers of iron homeostasis that were reported to be related to time to clinical worsening, mortality, and general outcome of patients with PAH, i.e., red cell distribution width and hypochromic erythrocytes, which might be beneficial in everyday practice
- Further investigations in the field of ID in pulmonary vasculature and PH are desired so as to enable the preparation of meta-analyses

## Figures and Tables

**Table 1:** PH—classification based on the hemodynamics.

	Pre-Capillary PH	Isolated Postcapillary PH	Mixed Pre- and Postcapillary PH
mPAP	>20 mmHg	>20 mmHg	>20 mmHg
PAWP	≤15 mmHg	>15 mmHg	>15 mmHg
PVR	>2 WU	≤2 WU	>2 WU

mPAP: mean pulmonary artery pressure, PAWP: pulmonary artery wedge pressure, PH: pulmonary hypertension, PVR: pulmonary vascular resistance, WU: Wood units.

**Table 2:** Five most commonly used definitions of ID.

ID 1—Strict Definition	ID 2—Liberal Definition	ID 3	ID 4	ID 5
Serum ferritin < 30 μg/L and TSAT < 16%	Serum ferritin < 100 μg/L and TSAT < 20%	Serum ferritin < 100 μg/L or ferritin 100–299 μg/L and TSAT < 20%	sTfR > 4.5 for women and sTfR > 5.0 for men	sTfR-F index > 3.2 if CRP < 5 mg/L or sTfR-F index > 2 if CRP > 5 mg/L

CRP: C-reactive protein, sTfR: soluble transferrin receptor, sTfR-F index: soluble transferrin receptor to log ferritin ratio, TSAT: transferrin saturation.

**Table 3:** The presentation of six studies concerning intravenous iron restoration in LHF.

Clinical Trial	Study Design	Sample Size	Iron Formula	Outcome
FAIR-HF	randomized, double-blind, placebo-controlled	459 HFrEF patients	Ferric carboxymaltose	Improvement in 6MWD, symptom burden and quality of life, moderate improvement in NYHA
HEART-FID	randomized, double-blind, placebo-controlled	3065 HFrEF patients	Ferric carboxymaltose	No apparent difference in death, hospitalizations related to HF or 6MWD
CONFIRM-HF	randomized, double-blind, placebo-controlled	304 HFrEF patients	Ferric carboxymaltose	Decreased risk of hospitalization due to HF, improved functional capacity (6MWD), symptoms burden and quality of life
FAIR-HF2	randomized, double-blind, placebo-controlled	1105 HFrEF patients	Ferric carboxymaltose	Neither apparent reduction in time to first HF hospitalization or cardiovascular death, nor decrease in the total number of HF hospitalizations. However, there was improvement in quality of life, symptom severity and functional capacity
AFFIRM-AHF	randomized, double-blind, placebo-controlled	1132 HFrEF patients	Ferric carboxymaltose	Study presents with reduced total hospitalizations due to HF, improved quality of life and functional capacity.
IRONMAN	randomized, open-label, blinded-endpoint	1137 HFrEF patients	Ferric derisomaltose	Lower risk of HF hospitalizations, improved quality of life

6MWD: 6-min walk distance, HF: heart failure, HFrEF: heart failure with reduced ejection fraction, NYHA: New York Heart Association scale.

**Table 4:** The comparison of the benefits and drawbacks of oral and intravenous routes of iron administration.

	Oral Route of Administration	Intravenous Route of Administration
Advantages	Cost-effective, convenient	Rapid and effective restoration; A suitable way to circumvent the possibility of impaired absorption
Disadvantages	Not effective enough, slow restoration, absorption may be disturbed due to the increased permeability of intestines, reduced gastric acid secretion, altered intestinal flora, frequent gastrointestinal side effects	Higher odds of hypersensitivity reactions, risk of iron overload, hyperviscosity symptoms and hypercoagulable state

**Table 5:** The presentation of studies concerning iron supplementation in PH patients.

Investigators	Study Sample	Iron Formula	Route of Administration	Duration	Outcome
Ruiter et al. [70]	21 iPAH patients	200 mg of ferrous fumarate	oral	4 weeks of treatment	There was an overall remarkable increase in ferritin levels, yet 44% of patients did not present with improved iron homeostasis
Ghio et al. [90]	22 PAH patients	30 mg of pyrophosphate sucrosomial iron	oral	16 weeks of treatment	There was reported significant improvement in 6MWD.
Olsson et al. [91]	22 PH patients	30 mg of ferric maltol	oral	12 weeks of treatment	There was observed increase in 6MWD and decrease in NT-proBNP values.
Blanche et al. [53]	142 cyanotic patients of whom 126 presented with PAH	FCM	intravenous	Median follow-up 100 days	Iron supplementation was related to improved iron status in these individuals.
Viethen et al. [73]	20 PAH patients; control group – PAH without ID	FCM	intravenous	2 months follow-up	Significant increase in fields of exercise capacity (6MWD) and quality of life was reported.
Kramer et al. [88]	117 PAH patients (of whom 58 were ID and 59 served as a control)	FCM	intravenous	18 months of observation	Remarkable improvement in 6MWD, WHO FC, ESC/ERS risk, decrease in number of hospitalizations was presented.
Biener et al. [89]	85 PH patients (31 of whom were ID, remaining 54 control group)	FCM	intravenous	16 weeks follow-up	There was an overall improvement in fatigue, however, the 3rd type of PH was the only one which presented with significant improvement in terms of 6MWD and WHO FC
Ruiter et al. [93]	15 iPAH patients	FCM	intravenous	12 weeks follow-up	Iron homeostasis was claimed to be improved, yet exercise capacity (6MWD) was unaltered. However, the exercise endurance time and aerobic capacity increased after FCM infusion.
Howard et al. [92]	39 iPAH/hPAH patients in Europe, 17 iPAH/hPAH patients in China	FCM in Europe; Iron dextran in China	intravenous	12 weeks follow-up	In spite of improvement in iron status, there was not observed any remarkable change in 6MWD or haemodynamics.

6MWD: 6-min walk distance, ERS: European Respiratory Society, ESC: European Society of Cardiology, FCM: ferric carboxymaltose, iPAH: idiopathic pulmonary arterial hypertension, hPAH: hereditary pulmonary arterial hypertension, NT-proBNP: N-terminal pro-B-type natriuretic peptide, PAH: pulmonary arterial hypertension, PH: pulmonary hypertension, WHO FC: WHO functional class.

## Data Availability

No new data were created or analyzed in this study.

## Abbreviations

The following abbreviations are used in this manuscript:

6MWD	6-min walk distance
CHD	Congenital Heart Disease
CPET	Cardiopulmonary exercise test
EPO	Erythropoietin
ES	Eisenmenger Syndrome
ESC	European Society of Cardiology
ET-1	Endothelin-1
HF	Heart failure
HIF	Hypoxia inducible factor
ID	Iron deficiency
IL-1	Interleukin-1
IL-6	Interleukin-6
mPAP	Mean Pulmonary Arterial Pressure
NO	Nitric Oxide
PAH	Pulmonary Arterial Hypertension
PASP	Pulmonary Arterial Systolic Pressure
PAWP	Pulmonary Artery Wedge Pressure
PGI_2_	Prostacyclin
PH	Pulmonary hypertension
PVR	Pulmonary Vascular Resistance
RHC	Right heart catheterization
sTfR	Soluble transferrin receptor
TSAT	Transferrin saturation
WU	Wood Units
